# Determinants of Protein Folding Pathways: Lessons from Metamorphic Proteins

**DOI:** 10.3390/ijms27104450

**Published:** 2026-05-15

**Authors:** Valeria Pennacchietti, Mariana Di Felice, Julian Toso, Laura Caldarelli, Eduarda Santos Ventura, Francesca Malagrinò, Angelo Toto, Stefano Gianni

**Affiliations:** 1 Laboratory Affiliated to Istituto Pasteur Italia, Fondazione Cenci Bolognetti, Dipartimento di Scienze Biochimiche “A. Rossi Fanelli”, Sapienza Università di Roma, 00185 Rome, Italy; valeria.pennacchietti@uniroma1.it (V.P.); mariana.difelice@uniroma1.it (M.D.F.); julian.toso@uniroma1.it (J.T.); laura.caldarelli@uniroma1.it (L.C.); eduarda.santosventura@uniroma1.it (E.S.V.); angelo.toto@uniroma1.it (A.T.); 2 Dipartimento di Medicina Clinica, Sanità Pubblica, Scienze della Vita e dell’Ambiente, Università dell’Aquila, Piazzale Salvatore Tommasi 1, Coppito, 67010 L’Aquila, Italy; francesca.malagrino@univaq.it

**Keywords:** kinetics, topology, mutagenesis, pathways

## Abstract

The protein folding problem has traditionally been defined by two complementary challenges: predicting the three-dimensional structure of a protein from its amino acid sequence and understanding the mechanism by which this structure is attained. While recent advances in artificial intelligence have largely addressed the former, the latter remains unresolved. Early studies showed that many small proteins fold in a cooperative two-state manner, shifting attention toward transition states and energy landscapes. Comparative analyses of protein families further revealed that folding mechanisms are often conserved among proteins sharing the same topology, suggesting a dominant role of structure in shaping folding pathways. However, this framework does not explain when and how a protein commits to a specific topology. Metamorphic proteins, in which highly similar sequences adopt distinct native folds, provide a powerful complementary approach. Studies of these systems show that closely related sequences can follow different folding mechanisms without sharing common intermediates. These findings indicate that folding pathways are determined at very early stages and are encoded within the denatured ensemble through subtle structural and energetic biases. Here, we review the evolution of protein folding studies and propose a unified view in which folding mechanisms are selected early, with the denatured state playing a central role in defining both folding pathways and final topology.

## 1. Introduction

The ability of proteins to fold into well-defined three-dimensional structures is a fundamental requirement for life. For decades, the protein folding problem has occupied a central position in molecular biology, biophysics, and structural biology, reflecting both its conceptual elegance and its biological importance [1,2,3,4,5,6]. Traditionally, this problem has been viewed as two closely related, yet distinct, challenges. The first concerns the prediction of the structure of a given protein from its amino acid sequence, while the second focuses on elucidating the mechanism by which a polypeptide chain navigates its conformational landscape to reach that structure.

Recent advances in artificial intelligence have profoundly transformed the first of these challenges. Structure prediction, once considered one of the most difficult problems in biology due to challenges such as conformational sampling, energy function accuracy, and the treatment of protein dynamics and disorder, has been largely addressed through the development of highly accurate computational tools capable of inferring protein structures directly from sequence information [7,8,9,10]. As a consequence, the emphasis of the field is increasingly shifting toward the second aspect of the folding problem, namely, understanding how proteins fold [11]. Despite decades of experimental and theoretical work, the detailed sequence of events that governs the transition from the denatured ensemble to the native state remains incompletely understood. This gap in knowledge is particularly striking given that folding is not only essential for biological function, but also intimately linked to misfolding and aggregation processes underlying numerous human diseases.

Early models of protein folding envisioned a largely sequential process in which elements of secondary structure, such as α-helices, β-sheets, and turns, form first, followed by the progressive assembly of tertiary interactions, including hydrophobic contacts, hydrogen bonds, salt bridges, and van der Waals interactions, through a series of partially structured intermediates [12]. This framework suggested a stepwise accumulation of native-like features, with folding proceeding along defined pathways populated by discrete species. However, this view was fundamentally challenged by the discovery that many small, single-domain proteins fold in an apparently cooperative, all-or-none manner [13]. In these systems, folding reactions can often be described as two-state processes, where only the denatured and native states are significantly populated, and no stable intermediates accumulate. This realization marked a turning point in the field, shifting attention from the identification of intermediates to the characterization of transition states and the underlying energy landscapes that govern folding.

The emergence of two-state folding raised important questions about the generality of folding mechanisms and the extent to which they are dictated by sequence or structure. To address these issues, considerable effort was devoted to the comparative study of protein families sharing a common fold. By analyzing proteins with similar three-dimensional structures but differing amino acid sequences, it became possible to isolate the role of topology in shaping folding pathways [14,15,16]. A major outcome of these studies was the observation that proteins adopting the same fold often exhibit remarkably conserved folding mechanisms, despite significant sequence divergence [15,17,18,19]. This finding suggested that native topology plays a dominant role in determining the folding process. However, sequence variations can modulate not only stability and kinetics but, in some cases, also the apparent folding pathway and cooperativity, particularly when they reshape the underlying energy landscape [20].

While the study of homologous proteins has provided invaluable insights [21,22,23,24,25,26], it is inherently limited by its focus on systems that converge to the same final structure. As such, it does not directly address a more fundamental question: at what stage of the folding reaction is the native topology selected? In other words, is the folding pathway determined progressively as structure forms, or is it encoded early in the denatured state, before any significant native-like features emerge? Addressing this question requires a different experimental strategy, one that decouples sequence from structure and allows direct comparison of alternative folding outcomes within closely related systems.

Metamorphic proteins offer a unique opportunity to tackle this challenge. These proteins, defined by their ability to adopt multiple distinct native folds from the same or highly similar amino acid sequences, provide an unprecedented framework to probe the determinants of folding pathways. In contrast to traditional protein families, where different sequences lead to the same structure, metamorphic systems invert the problem: nearly identical sequences give rise to different topologies [27,28,29,30]. This property makes them particularly powerful for dissecting the relationship between sequence, structure, and folding mechanism. Importantly, both naturally occurring and engineered metamorphic proteins have been identified, with the latter offering highly controlled systems in which minimal sequence changes can trigger dramatic structural rearrangements [31,32].

The study of metamorphic proteins has led to a fundamental insight: folding mechanisms can diverge at very early stages of the reaction, and distinct native structures may be encoded within the denatured ensemble itself [33,34]. Rather than converging through shared intermediates, proteins with similar sequences can follow entirely different pathways, reflecting subtle energetic biases that predefine their folding trajectories. These observations challenge early stepwise models of protein folding that emphasized the sequential accumulation of structure through discrete intermediates and instead support a description in which folding occurs on a pre-shaped energy landscape. In this framework, topology selection emerges early, and the subsequent folding process refines and consolidates this initial bias.

In this review, we will examine how the field has evolved from the early characterization of folding kinetics to the modern understanding of energy landscapes and folding mechanisms. We will first revisit the historical development of protein folding studies, focusing on the discovery of two-state folding and the experimental approaches used to identify and characterize intermediates. We will then discuss the insights gained from the study of protein families, highlighting the role of topology in shaping conserved folding pathways. Finally, we will explore the emerging perspective offered by metamorphic proteins, emphasizing their contribution to redefining our understanding of how folding mechanisms are determined and how structural diversity can arise from minimal sequence variation.

## 2. The Historical Development of Folding Kinetics: From Sequential Models to Two-State Folding

The earliest attempts to describe protein folding were strongly influenced by the intuitive notion that structure forms progressively through a sequence of well-defined steps [35,36,37]. In this classical view, folding was envisioned as a hierarchical process in which local elements of secondary structure, such as α-helices and β-sheets, form first and subsequently assemble into the native tertiary structure. This framework naturally implied the existence of partially structured intermediates, each representing a distinct stage along the folding pathway [38,39]. For many years, the identification and characterization of such intermediates became a central objective in the study of protein folding [12].

Experimental support for this stepwise view initially emerged from studies employing spectroscopic techniques capable of monitoring structural changes during folding reactions. These experiments often revealed complex kinetics that were interpreted in terms of multiple populated states. Although rapid mixing techniques such as stopped-flow spectroscopy were already available, their systematic application to protein folding brought kinetic measurements to the forefront of the field. This shift enabled the quantitative characterization of folding reactions with high temporal resolution and led to a reinterpretation of folding behavior. These advances led to a surprising and transformative realization: many small, single-domain proteins fold in an apparently cooperative manner, without detectable accumulation of intermediate species [13,40], with the first protein to be identified as a two-state folder being the Chymostrypsin inhibitor 2 [13].

This behavior, termed two-state folding, implies that the protein exists predominantly in either the denatured or the native state, with a single free-energy barrier separating the two. Under such conditions, folding and unfolding kinetics are typically described by single-exponential time courses, reflecting the absence of stable intermediates along the reaction coordinate [13]. The discovery of two-state folding challenged the prevailing paradigm and suggested that, at least for a large class of proteins, the folding process is far simpler than previously assumed. Rather than proceeding through a series of discrete steps, folding could occur in a highly concerted fashion, with the majority of native interactions forming cooperatively as the protein crosses the transition state barrier [13,40].

The recognition of two-state behavior shifted the focus of the field from the detection of intermediates to the characterization of the transition state, a high-energy, transient configuration that cannot be directly observed but plays a central role in determining folding kinetics [41,42,43,44,45]. Because the transition state does not accumulate, its structural features must be inferred indirectly. This challenge led to the development of powerful experimental approaches, most notably mutational analyses that probe the effects of specific perturbations on folding and stability. By systematically modifying individual residues and measuring the resulting changes in folding rates and thermodynamic stability, it became possible to extract residue-level information about the degree of native-like structure present in the transition state, with an invaluable experimental technique named the Φ value analysis [44,46,47].

These studies contributed to the formulation of general mechanistic models of protein folding, among which the nucleation–condensation mechanism has emerged as one of the most influential [41,48,49]. In this framework, folding is initiated by the formation of a small, partially organized nucleus, which stabilizes the subsequent collapse and consolidation of the entire polypeptide chain. Importantly, nucleation and global compaction are not strictly sequential events but occur in a coupled manner, giving rise to a transition state that already exhibits significant native-like features, albeit in a distorted and dynamic form. This model, which emerged both from experimental [44,48] and computational [41] work, reconciles the apparent simplicity of two-state folding with the underlying complexity of the interactions that drive it, emphasizing the cooperative nature of the process.

Despite the success of the two-state paradigm, it soon became clear that not all proteins conform to this behavior. Larger proteins, multidomain systems, or proteins under specific environmental conditions often display more complex folding kinetics, characterized by the presence of one or more intermediate states [50,51,52]. In such cases, deviations from simple two-state behavior can be detected through characteristic features in kinetic experiments, such as curvature in chevron plots or the presence of multiple kinetic phases [53,54,55]. These observations indicate that intermediates, whether on-pathway or off-pathway, can play a significant role in shaping the folding landscape.

The structural characterization of folding intermediates has therefore remained an important objective, providing insights into the sequence of events that occur during folding and the factors that stabilize partially folded states, as well as in highlighting their putative cryptic functions [56,57]. Advances in experimental techniques, including time-resolved spectroscopy, hydrogen–deuterium exchange, and single-molecule methods, have enabled the detection and description of these transient species with increasing precision [58,59,60]. In parallel, computational approaches have contributed to mapping the conformational landscapes accessible to proteins and identifying potential intermediate states [61,62,63,64].

Taken together, the historical development of folding kinetics reveals a field that has progressively moved from a deterministic, stepwise view of folding toward a more nuanced understanding based on energy landscapes and cooperative transitions. The recognition that proteins can fold via both simple two-state mechanisms and more complex pathways involving intermediates underscores the diversity of folding behaviors and highlights the importance of identifying the factors that determine which mechanism a given protein will follow. This question, what dictates the folding pathway, remains central and sets the stage for the approaches discussed in the following sections.

## 3. Protein Families and the Conservation of Folding Mechanisms

One of the major challenges in the study of protein folding lies in the intrinsic difficulty of comparing different systems. Proteins vary widely in their amino acid sequences, native structures, and even in the properties of their denatured states. As a result, drawing general conclusions from the analysis of unrelated proteins is often problematic, as observed differences in folding behavior may arise from multiple, confounding factors. To overcome this limitation, the field progressively converged on a more controlled experimental strategy: the comparative study of proteins belonging to the same structural family [15,17,18,19,65,66].

Protein families provide an ideal framework for isolating the determinants of folding mechanisms. Members of a given family typically share a common three-dimensional fold while displaying varying degrees of sequence divergence. This combination allows researchers to probe how changes in sequence affect folding behavior without altering the overall topology of the native state. By minimizing structural variability, it becomes possible to focus on the relationship between sequence and folding kinetics, and to assess the extent to which folding pathways are conserved.

**Figure 1:**
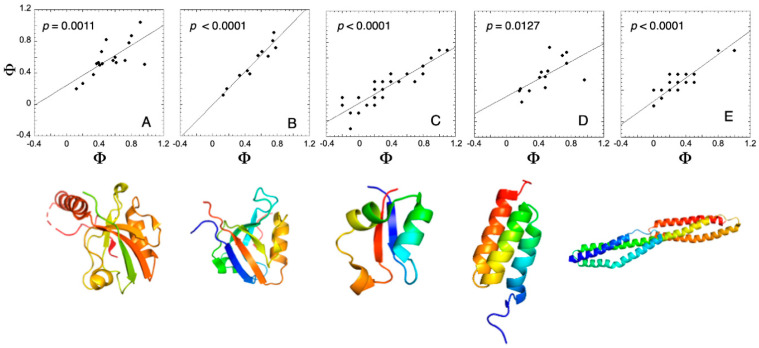
Φ–Φ plots across protein systems with different topologies. Φ–Φ plots are shown for a set of proteins differing in sequence, connectivity, and structural organization. Each panel reports a distinct system, including proteins from the same superfamily (**A**), circular permutants (**B**), circularized variants (**C**), and constructs with altered domain organization (**D**) or probing methods (**E**). The corresponding three-dimensional structures are displayed below each plot to highlight differences in topology. Despite these variations, all datasets exhibit a consistent linear relationship, as indicated by the best-fit lines. The reported *p*-values quantify the probability that the two variables are not correlated, supporting the robustness of the observed relationship across diverse protein topologies.

A large body of work based on this approach has revealed a striking and somewhat unexpected result: proteins that adopt the same fold often display highly similar folding mechanisms, even when their sequences differ substantially [15,17,18,19,65,66]. This observation has been consistently supported by kinetic analyses, mutational studies, and comparisons of transition state structures [67,68]. In many cases, the distribution of native-like interactions in the transition state, as inferred from residue-specific perturbations, is conserved across homologous proteins. Such findings indicate that the folding pathway is largely dictated by the topology of the native structure, with sequence variations primarily modulating stability and folding rates rather than fundamentally altering the mechanism. Figure 1 exemplifies the conservation of the observed Φ values in proteins displaying similar topologies but different sequences (A, [69]), in a protein with one of its corresponding circular permutants (B, [70]), a protein and its circularized variant (C, [71]), a protein characterized by different optical probes (D, [72]) and a protein in the presence/absence of a contiguous folded domain (E, [73]). The structures of the different cases are also shown in the figure together with the relevant references of the respective mutational works.

This concept is closely linked to the idea that protein folding is governed by the underlying energy landscape, which is shaped by the arrangement of native contacts [5,74,75,76]. Proteins sharing the same topology are expected to possess similar energy landscapes, characterized by comparable distributions of energetic barriers and transition states. Consequently, the folding reaction tends to proceed through analogous pathways, regardless of the specific amino acid composition. In this view, sequence plays a secondary role, fine-tuning the energetic details of the landscape without redefining its overall architecture.

The conservation of folding mechanisms within protein families has important implications. First, it suggests that general principles of folding can be extracted by studying a limited number of representative systems, provided that they are carefully chosen. Second, it supports the notion that topology is a primary determinant of folding behavior, reinforcing the idea that the native structure encodes not only the final state but also the pathway by which it is reached [45]. Finally, it provides a framework for interpreting the effects of mutations, as changes in sequence can be understood in terms of their impact on a largely conserved folding mechanism.

However, despite its success, this approach is inherently limited in scope. By focusing on proteins that converge to the same native structure, it implicitly assumes that the folding pathway is determined by the final topology. As a result, it cannot directly address whether alternative folding mechanisms might exist for closely related sequences, or at what stage of the folding process a protein commits to a specific topology. In other words, while studies of protein families demonstrate that folding mechanisms are conserved when the structure is conserved, they do not reveal how or when this structural outcome is selected.

This limitation highlights the need for complementary systems in which sequence and structure can be decoupled. To fully understand the determinants of folding pathways, it is necessary to investigate proteins that challenge the one-sequence–one-structure paradigm and allow direct comparison of alternative folding outcomes within closely related sequences. It is precisely in this context that metamorphic proteins emerge as a powerful and informative class of systems, as discussed in the following section.

## 4. Metamorphic Proteins as a Complementary Approach to Protein Folding

While the study of protein families has provided compelling evidence that folding mechanisms are largely conserved among proteins sharing the same topology, this approach is intrinsically constrained by its focus on systems that converge to a single native structure. As such, it cannot directly address a fundamental question: at what stage of the folding reaction is the native topology selected? To tackle this issue, it is necessary to adopt a complementary strategy in which the relationship between sequence and structure is decoupled. Metamorphic proteins provide precisely such an opportunity.

Metamorphic proteins are defined by their ability to adopt multiple distinct native conformations from the same or highly similar amino acid sequences [28,29,30,31,32]. In contrast to traditional protein families, where different sequences fold into the same structure, metamorphic systems invert the problem: nearly identical sequences give rise to different topologies [77,78,79]. This unique property makes them particularly powerful tools for probing the determinants of protein folding, as it allows one to isolate the factors that drive structural divergence while minimizing sequence variability.

**Figure 2:**
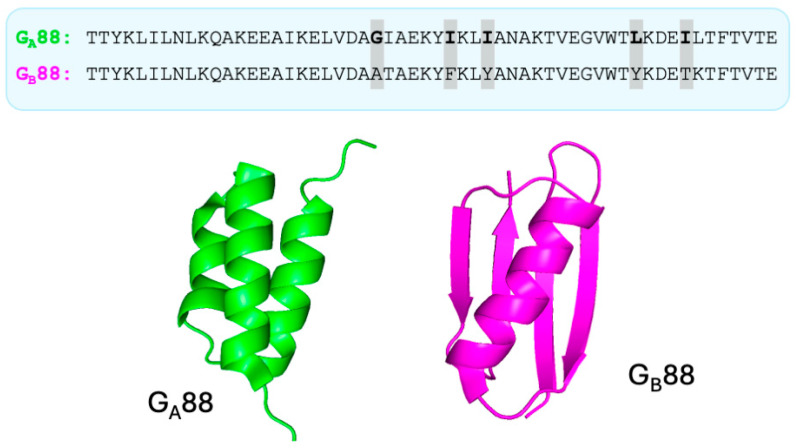
Sequence and structure of G_A_88 and G_B_88. Amino acid sequences of G_A_88 (green) and G_B_88 (magenta) are shown at the top, with differing residues highlighted. Despite their high sequence similarity, the two proteins adopt distinct folds, as illustrated by their three-dimensional structures below. G_A_88 forms an α-helical bundle, whereas G_B_88 adopts an α/β fold. This comparison highlights how limited sequence variations can give rise to markedly different topologies.

Although examples of naturally occurring metamorphic proteins have been identified, recent advances in protein design have enabled the creation of highly controlled model systems in which fold switching can be studied with remarkable precision [27,28,30]. These engineered proteins often differ by only a handful of residues, or even a single amino acid substitution, yet they populate distinct structural states (see, for example, the case reported in Figure 2). In some cases, variants can be generated that interconvert between folds or simultaneously populate multiple conformations, providing a direct window into the energetic balance between competing structural minima [27,28].

The key advantage of metamorphic proteins lies in their ability to reveal how subtle energetic differences influence folding pathways [80]. Because the sequences involved are nearly identical, any divergence in folding behavior must arise from minimal perturbations that reshape the underlying energy landscape [81]. This allows one to ask whether different folds are reached through common intermediates or whether distinct pathways are selected from the outset of the folding reaction. In this respect, metamorphic proteins provide a level of resolution that is difficult to achieve with traditional comparative approaches.

**Figure 3:**
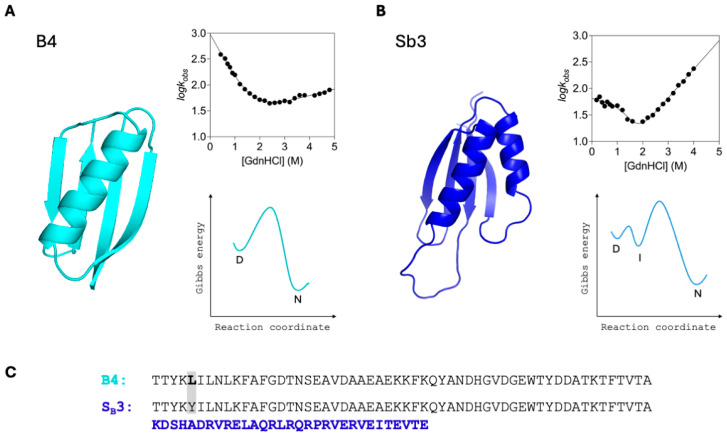
Folding behaviour of B4 and Sb3. (**A**) B4 (cyan) displays two-state folding behavior, as indicated by the characteristic V-shaped chevron plot (log k_obs_ versus denaturant concentration), consistent with a single transition between denatured (D) and native (N) states. The corresponding free energy profile shows a single barrier separating D and N. (**B**) Sb3 (blue) exhibits three-state folding, as evidenced by curvature in the chevron plot, indicating the presence of an intermediate (I). The associated energy landscape reveals multiple barriers corresponding to D, I, and N states. Chevron plots report the logarithm of the observed folding/unfolding rates (k_obs_) as a function of denaturant concentration, allowing discrimination between simple two-state and more complex folding mechanisms. (**C**) Sequence alignment of B4 and Sb3, with differing residues highlighted, showing that modest sequence variations give rise to distinct folding mechanisms.

A compelling experimental illustration of these principles is provided by the comparative study of the engineered metamorphic proteins B4 and Sb3 [82]. Despite sharing nearly identical sequences over a substantial portion of their length, these proteins adopt distinct topologies and, strikingly, follow different folding mechanisms. As shown in Figure 3, B4 displays a classical two-state folding behavior, characterized by a V-shaped chevron plot and a single free-energy barrier separating denatured and native states. In contrast, Sb3 exhibits a pronounced curvature in the chevron plot, indicative of a three-state mechanism involving the accumulation of a folding intermediate, which is also reflected in a more complex energy landscape. These results demonstrate that even minimal sequence differences, when coupled to changes in topology, can lead to qualitatively different folding pathways, supporting the idea that the selection of the folding route is intimately linked to the final structural organization. Remarkably, the same work showed that mutation of Y 5 into L in Sb3 corresponds to the population of both structures simultaneously and returns double exponential folding kinetics. The two phases correspond to those observed for B4 and Sb3, strongly supporting the robustness of topology-defined folding mechanisms [82].

Studies of these systems have led to a striking conclusion: proteins with highly similar sequences can follow fundamentally different folding mechanisms [33,34,82], and these differences emerge at very early stages of the reaction. Rather than converging through shared intermediates, metamorphic proteins often display distinct kinetic behaviors, with some variants folding via simple two-state mechanisms and others populating intermediate species. Importantly, the intermediates observed in one folding pathway do not necessarily correspond to alternative native structures, indicating that different folds are not accessed through a common structural precursor.

These observations strongly support the notion that the folding pathway is not determined solely by the progressive accumulation of native-like structure, but is instead encoded in the initial conditions of the system [83,84,85,86,87]. In particular, the denatured state, often shallowly assumed as a relatively featureless ensemble, appears to contain residual structural biases that predispose the protein toward a specific topology [88]. Even subtle differences in sequence can modulate these biases, shifting the balance between competing folding routes and leading to the selection of distinct native states [33,82,89].

From the perspective of energy landscape theory, metamorphic proteins can be viewed as systems in which multiple competing minima are accessible within a relatively shallow landscape. Small perturbations in sequence or environmental conditions can therefore reshape the landscape, altering the relative stability of these minima and the pathways connecting them. In this framework, folding is not a simple downhill process toward a unique global minimum, but rather a competition between alternative structural outcomes, each associated with its own kinetic route.

The implications of these findings are profound. They challenge the traditional view that folding pathways are largely determined by the final structure and instead suggest that topology is selected at a very early stage of the folding reaction. Moreover, they indicate that different native states can be encoded within closely related sequences without the need for extensive structural rearrangements along a common pathway. In this sense, metamorphic proteins reveal a previously underappreciated level of plasticity in protein folding, highlighting the importance of early energetic biases and residual structure in shaping folding mechanisms.

In the following section, we will examine how these insights converge into a unified picture in which folding pathways are determined at the level of the denatured state, leading to the early commitment of proteins to specific topologies and the absence of shared intermediates between alternative folds.

## 5. Early Determination of Folding Pathways: Commitment in the Denatured State

The insights gained from the study of metamorphic proteins converge toward a unifying and conceptually powerful conclusion: the folding pathway of a protein is determined at a very early stage of the reaction, and the choice of native topology is encoded within the denatured state. This perspective represents a significant departure from traditional views of protein folding, which often assume that structure emerges progressively along the reaction coordinate through the accumulation of native-like interactions.

In classical models, the denatured state is frequently treated as a relatively featureless ensemble, lacking persistent structural elements and serving primarily as a starting point for folding [12,35,36,37,38,39]. Within this framework, the formation of intermediates and the progressive stabilization of native contacts are thought to guide the protein toward its final structure [36,54,90,91]. However, the behavior of metamorphic proteins challenges this assumption, suggesting instead that the denatured ensemble may already contain subtle structural and energetic biases that predefine the folding trajectory.

A central observation supporting this view is the absence of common intermediates between alternative folds adopted by closely related sequences. If folding proceeded through shared structural precursors, one would expect different variants of a metamorphic system to converge, at least transiently, onto similar intermediate states before diverging toward their respective native conformations. Instead, experimental evidence indicates that distinct folding pathways are selected from the outset, with different variants exhibiting fundamentally different kinetic mechanisms [33,34,82]. In some cases, one protein may fold via a simple two-state process, while a closely related variant populates one or more intermediate states, without any indication that these intermediates correspond to alternative native folds [82].

This lack of shared intermediates implies that the divergence between folding pathways occurs before the formation of any stable, partially folded species [92]. In other words, the commitment to a specific topology must take place at an early stage of the folding reaction, likely within the denatured ensemble itself [34]. Even though this ensemble is highly dynamic and heterogeneous, it is not structurally random. Instead, it appears to be biased by residual interactions—such as transient secondary structure, hydrophobic clustering, or long-range contacts—that influence the accessibility of different regions of the energy landscape.

These residual structural features can be highly sensitive to small perturbations in sequence or environmental conditions. In metamorphic systems, minimal changes, such as single amino acid substitutions or variations in chain length, are sufficient to alter the balance between competing structural states [89]. Such perturbations can modulate the population of specific conformations within the denatured ensemble, effectively steering the folding process toward one topology over another [93]. Once this early bias is established, the subsequent folding reaction proceeds along a pathway that is largely consistent with the selected topology, without requiring large-scale rearrangements or transitions between alternative folds [94].

From the perspective of energy landscape theory, these observations suggest that the folding landscape is partitioned into distinct basins corresponding to different native states, and that the entry point into one basin or another is determined very early during the folding process. Rather than exploring the entire conformational space and subsequently selecting the most stable structure, the protein is effectively “guided” into a specific region of the landscape by the intrinsic properties of its denatured state. This early partitioning reduces the need for interconversion between competing structures and explains the absence of common intermediates.

An important implication of this model is that folding mechanisms are not solely dictated by the final native structure, but also by the initial conditions from which folding begins [68,95]. While topology remains a dominant factor in shaping the overall energy landscape, the pathway by which that topology is reached is influenced by subtle features of the denatured ensemble. This interplay between early structural biases and global topology provides a more nuanced understanding of protein folding, in which both sequence-dependent and topology-dependent factors contribute to the determination of folding mechanisms [34,84,87,96].

Taken together, these findings support a view of protein folding in which the decisive events occur at the very beginning of the reaction. The denatured state is not merely a passive starting point, but an active determinant of folding pathways, encoding the information necessary to select between alternative topologies [97,98]. In this framework, the folding process can be seen as the amplification of initial biases rather than the gradual construction of structure, with early decisions shaping the entire trajectory of the reaction. This perspective not only reconciles observations from metamorphic proteins with established principles of folding but also provides a conceptual foundation for understanding how structural diversity can arise from minimal sequence variation.

## 6. Implications and Future Perspectives

The view that folding pathways are determined at the level of the denatured state has important implications for our understanding of protein folding, function, and evolution. By shifting attention from the progressive formation of structure to the role of intrinsic energetic biases, this perspective provides a unifying framework that integrates insights from folding kinetics, protein families, and metamorphic systems.

A natural connection emerges with the concept of templated folding, in which interaction partners guide folding by shaping the transition state and directing the pathway [99,100,101]. This behavior highlights that pathway selection is not solely encoded in the amino acid sequence, but can also be influenced by external interactions that bias the energy landscape [102,103]. In this context, metamorphic proteins represent an intrinsic counterpart, where alternative folding pathways arise from competing basins within the same sequence, suggesting that similar principles operate even in the absence of a binding partner. These observations are closely linked to the concept of energetic frustration [104,105,106]. While classical folding theory emphasizes minimally frustrated, funneled landscapes, residual frustration introduces competing interactions and alternative minima, enabling multiple folding routes and structural outcomes [75,104,105,106,107,108]. Metamorphic proteins exemplify this behavior, as subtle sequence variations reshape the balance between competing states and lead to distinct folding mechanisms. In this framework, pathway selection can be viewed as the resolution of frustrated interactions already present in the denatured ensemble.

Together, these perspectives suggest that folding mechanisms arise from a balance between intrinsic sequence-encoded biases, landscape frustration, and, in some cases, external templating effects [75]. This expanded view has important implications for protein evolution, as it implies that small perturbations can redirect folding pathways and enable the emergence of new structural and functional states without extensive sequence divergence.

From a methodological standpoint, these findings highlight the need for approaches capable of characterizing the denatured ensemble and its residual structure. Advances in spectroscopic methods, single-molecule techniques, and molecular simulations will be essential to directly probe the early determinants of folding pathways and to quantify the role of frustration and templating in shaping the energy landscape. Based on the evidence discussed, we propose that folding pathways are determined by sequence-encoded structural and energetic biases within the denatured ensemble, which partition the energy landscape at early stages and define the accessible topological basins prior to the formation of stable intermediates. This hypothesis predicts that perturbations affecting residual structure in the denatured state should systematically redirect folding mechanisms, providing a direct experimental route to test early pathway selection.

## Data Availability

No new data were created or analyzed in this study. Data sharing is not applicable to this article.
